# Algal Carotenoids: Chemistry, Sources, and Application

**DOI:** 10.3390/foods12142768

**Published:** 2023-07-20

**Authors:** Ivana Generalić Mekinić, Vida Šimat, Nikheel Bhojraj Rathod, Imen Hamed, Martina Čagalj

**Affiliations:** 1 Department of Food Technology and Biotechnology, Faculty of Chemistry and Technology, University of Split, R. Boškovića 35, HR-21000 Split, Croatia; 2 University Department of Marine Studies, University of Split, R. Boškovića 37, HR-21000 Split, Croatia; martina.cagalj@unist.hr; 3 Department of Post Harvest Management of Meat, Poultry and Fish, PG Institute of Post Harvest Technology & Management (Dr. Balasaheb Sawant Konkan Krishi Vidyapeeth, Dapoli), District Raigad, Killa-Roha 402 116, Maharashtra State, India; nikheelrathod310587@gmail.com; 4 Department of Biotechnology and Food Science, NTNU—Norwegian University of Science and Technology, 7491 Trondheim, Norway; imen.hamed@ntnu.no

**Keywords:** microalgae, *Chlorophyta*, *Phaeophyta*, *Rhodophyta*, carotenoids, isolation, detection, application

## Abstract

Recently, the isolation and identification of various biologically active secondary metabolites from algae have been of scientific interest, with particular attention paid to carotenoids, widely distributed in various photosynthetic organisms, including algal species. Carotenoids are among the most important natural pigments, with many health-promoting effects. Since the number of scientific studies on the presence and profile of carotenoids in algae has increased exponentially along with the interest in their potential commercial applications, this review aimed to provide an overview of the current knowledge (from 2015) on carotenoids detected in different algal species (12 microalgae, 21 green algae, 26 brown algae, and 43 red algae) to facilitate the comparison of the results of different studies. In addition to the presence, content, and identification of total and individual carotenoids in various algae, the method of their extraction and the main extraction parameters were also highlighted.

## 1. Introduction

Carotenoids are the most widely distributed lipid-soluble pigments in nature. They are common in various photosynthetic organisms (higher plants, fungi, algae, and bacteria), where they are primarily biosynthesized and are responsible for the various colors, hues, and shades [1]. They play several roles: during photosynthesis they act as accessory pigments for light collection, they are involved in preventing damage from excess light and serve as one of the most important cell antioxidants, they are important in the reproductive cycle as their color attracts pollinators, they are provitamins, etc. [2,3]. Carotenoids are a very complex and heterogeneous group of compounds whose greatest structural diversity is found in organisms of the marine environment. Some carotenoids are restricted exclusively to aquatic sources. Complex habitats and harsh conditions have led algae to produce a wide range of specific and bioactive compounds. Due to the diverse bioactivity of algae and their use in food, medicine, and cosmetics, they are currently being intensively studied for the production of carotenoid compounds [4,5,6,7].

**Figure 1:**
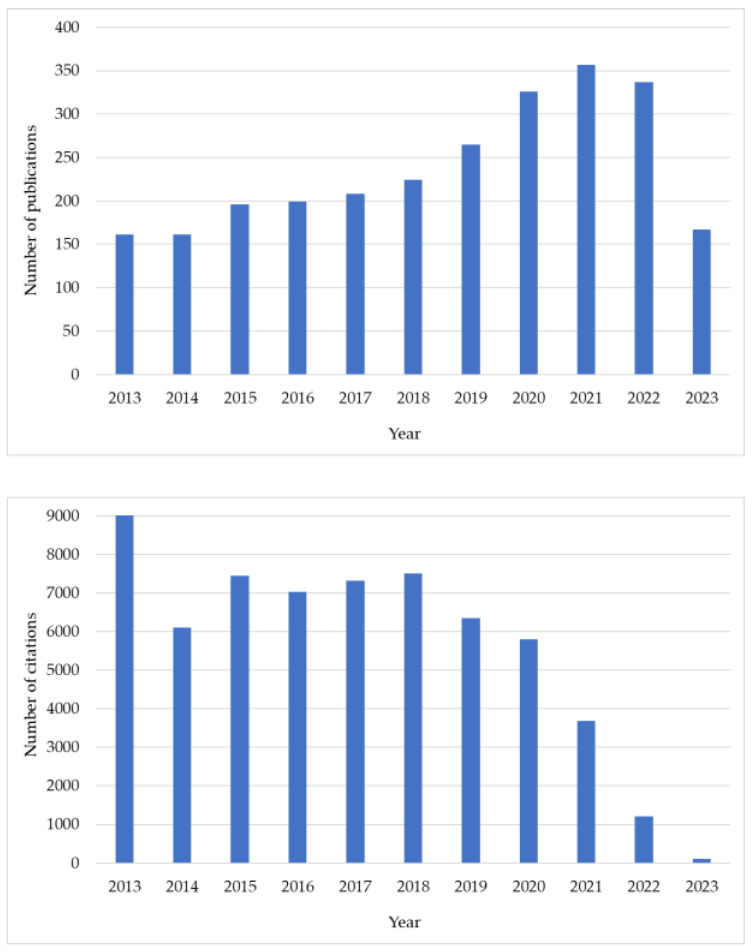
Number of publications and citations per year (until 23 May 2023) related to the carotenoids in algae. Data were obtained from Scopus with the following search criteria: Title, Abstract, Keyword; Carotenoids AND Algae.

Currently, commercially available carotenoids are usually produced by chemical synthesis, which involves high economic costs and has a negative impact on the environment. These facts have led to a significant increase in demand for natural sources of carotenoids, with a focus on marine organisms, especially algae. According to the Scopus database, the number of publications and citations related to carotenoids in algae has increased in the last decade, and it is still permanently increasing (Figure 1).

In this review, articles published from 2015 (via keyword searches in the Scopus and Science direct databases) on the presence, isolation, and detection of carotenoid compounds (total and individual) in various microalgal and macroalgal species (green, brown, and red algae and seaweeds) are summarized and discussed. Particular attention was paid to the extraction solvent and procedure used, as well as the method/technique used for detection and the concentrations reported.

## 2. Carotenoids

### 2.1. Chemistry

Chemically, carotenoids are isoprenoid polyenes. Their structure consists of isoprene (C5) units that are attached to each other, so we usually divide them into C30 (6 units, apocarotenoids), C40 (8 units), C45 (9 units), and C50 (10 units). However, the most abundant carotenoids in nature are the carotenoids with 40 C atoms (tetraterpenoids). Their molecule is symmetrical as the isoprenes are connected in a head-to-tail manner except in the center of the molecule, where the order is reversed (tail to tail). One can also distinguish carotenoids with allenic (C=C=C), and acetylenic (C≡C) groups, which are found in various marine organisms [5,8,9].

Carotenoids are generally classified as carotenes and xanthophylls. Carotenes are hydrocarbons (C_40_H_56_), while xanthophylls are their oxygenated derivatives that have one or more oxygen-containing functional groups, such as methoxy- (e.g., rhodovibrin and spirilloxanthin), hydroxy- (e.g., criptoxanthin, zeaxanthin, and lutein), keto- (e.g., astaxanthin and canthaxanthin), epoxy- (e.g., violaxanthin, neoxanthin, and fucoxanthin), carboxy- (e.g., bixin and crocetin), oxo- (e.g., capsanthin and rhodoxanthin), and ester (xanthophyll esterification with fatty acids) forms. Xanthophylls are usually formed from the basic structure by enzymatic reactions that undergo hydrogenation, dehydrogenation, cyclization, and oxidation, and the oxygen-containing functional groups in the xanthophyll structure affect their biological properties and solubility, making them more polar than carotenes [1,8,9,10]. They can also be classified as acyclic, monocyclic, and bicyclic, based on the number of molecular terminal rings [11].

Due to the presence of double bonds in the carotene molecule, they often exhibit *cis*-*trans* isomerization. In nature, however, carotenoids exist predominantly in *trans*- form. This structural feature also makes them susceptible to oxidation (autooxidation, photooxidation, and enzymatic oxidation) [8]. Carotenoids are mostly stable as long as the integrity of the cell and chromoplast is maintained. However, when this is disturbed, they are susceptible to the exposure to oxygen and oxidizing enzymes, light, elevated temperatures, the presence of metal ions, prooxidants, etc., leading to their degradation. Carotenoids are also important from a nutritional point of view. The chemical diversity of carotenoids leads to their various biochemical and physiological functions, such as antioxidant, anticancer, chemoprotective, lipotoxic, anti-inflammatory, neuroprotective, antidiabetic, etc. [2,3,9,10].

### 2.2. Extraction

The polyene chain in the structure of carotenoids is responsible for their characteristic yellow, orange, and red colors, while the nature of the oxygenated groups is responsible for their solubility, polarity, and general chemical properties. The extraction of liposoluble pigments from plant material usually involves the use of vegetable oils and various organic solvents [10].

Carotenes such as β-carotene, α-carotene, and lycopene are the most widely distributed nonpolar carotenoids, while the presence of polar functional groups in xanthophyles increases the polarity of these compounds [11].

Therefore, carotenoids are liposoluble: carotenes in nonpolar or medium-polar solvents (such as hexane, ethyl acetate, and petroleum ether) and xanthophylls in polar solvents such as alcohols or acetone [8,12]. In most studies, mixtures of acetone, ethanol/methanol, and hexane are used for the extraction of carotenoids from various matrices, but ethanol and acetone prove to be very efficient when large amounts of water are present, as in algal samples. In terms of safety and environmental protection, these two solvents are also the best choices for food applications [11,12,13,14,15].

Prior to extraction, the often dry algal samples are homogenized and then extracted with an organic solvent/solvent mixture, usually applied in one step. The choice of solvent is a critical factor for efficient carotenoid extraction. Since carotenoids are susceptible to degradation reactions and enzymatic oxidation, extractions should be performed in a short time and under suitable temperature conditions [11,12].

In line with the principles of environmentally friendly extraction, extraction processes have recently been developed that minimize the use of organic solvents, utilize renewable resources, and also ensure a high content of extracted compounds and their quality. Various methods have been developed for the rapid and efficient extraction of carotenoids from algae, usually combined with cell disruption to facilitate the release of carotenoid pigments [16,17,18,19]. Strategies involving mechanical disruption followed by solvent extraction have increased the yield of extracted carotenoid compounds [20,21,22]. However, considering the risks associated with mechanical cell disruption, enzymes have also been used as cell wall disruption technology [23]. Considering the risks associated with the use of harmful solvents and with advanced nonthermal technologies, these are also being investigated for the extraction of bioactive compounds [24], as novel technologies using high pressure, ultrasound, microwaves, pulsed electric fields, supercritical fluids, pressurized fluids, moderate electric fields, enzymes, etc. [11,12,17,25,26,27,28,29]. Recent reviews [16,18,20,30,31,32,33] compared the different conventional and novel methods for the extraction of carotenoids from algae, such as physical, chemical, mechanical (grinding, milling, pressure), microwave-assisted extraction, ultrasound-assisted extraction, enzymatic hydrolysis, application of electric fields, ionic liquids, deep eutectic solvents, chemical solvation, extraction with supercritical fluids, etc.

Carotenoid extraction often involves the extraction of other compounds such as chlorophylls, lipids, and esters, so saponification (alkaline hydrolysis) is an important step to remove these compounds that could interfere with the analysis. During saponification, the fatty acids bound to the target molecules are broken up and released. This process thus separates other lipid compounds, especially triglycerides, from the carotenoids. While carotenes do not form esters, xanthophylls do, so one of the main advantages of saponification is the release of esterified xanthophylls through the hydrolysis [11,34]. Also, this step is often used to remove interfering chlorophylls. From the above, it can be concluded that saponification is usually required for the hydrolysis of carotenoid-esterified forms (in samples containing xanthophyll esters), for the separation of chlorophylls, or in samples with high contents of lipids and low contents of carotenoids [34]. Saponification can be performed during sample homogenization, before or after extraction, and involves the addition of a basic solvent (e.g., methanolic sodium or potassium hydroxide). This step does not affect most carotenoids as they are alkali-stable, but some of them such as astaxanthin and fucoxanthin are susceptible to this reaction. It has also been reported that saponification leads to the degradation of carotenoids (isomerization, decomposition by high temperatures, long time and high alkali concentration, oxidation, etc.), so it should be omitted if possible [35,36].

### 2.3. Identification, Separation, and Quantification

Prior to quantification, carotenoids are usually identified by UV/Vis spectrophotometry (detection of absorption maxima at specific wavelengths), Fourier transform infrared spectroscopy (FTIR) (determination of the nature of functional groups), and nuclear magnetic resonance (NMR) (conjugation of double bonds) [37]. Nonspecific spectrophotometric methods are used for the rapid screening and determination of total carotenoid content in the samples, while chromatographic techniques are used for the separation, identification, and quantification of individual compounds [10,11,38].

Carotenoids are able to absorb ultraviolet (UV) and light in the visible region of the spectrum because they have conjugated double bonds in their structure (chromophore). They usually have three absorption maxima in the visible region of the spectrum (between 430 and 480 nm), and only a few of them have maxima in the UV region (e.g., phytoene). Therefore, the differences in the spectral characteristics of individual carotenoids are often small but very important for their identification. Carotenoid absorption maximums depend on the nature of the carotenoid (polyenic extremity, presence of the C=O conjugated groups, and the *cis*–*trans* configuration of the molecule). It should also be noted that the used solvent has a great influence on the absorption maximum of the compound [38,39,40]. The total carotenoid content of isolates is usually determined by measuring the absorbance of the sample at a specific wavelength and calculating it from the absorbance values reported in the literature. In this method, the content of total carotenoids is usually expressed as equivalents of β-carotene [8,10,11,37,39,40].

The separation and quantification of carotenoids is usually performed by high-performance liquid chromatography (HPLC) using different stationary phases (octyl-C8, octadecyl-C18, and C30), with C18 being the most commonly used and C30 being more efficient in separating the geometric isomers. Their separation can be performed by normal and reversed phase HPLC, but the separation of carotenes by normal phase HPLC is usually not good [41]. The most common detectors are ultraviolet (UV), visible (Vis), and diode array detector (DAD). In these methods, carotenoids are identified based on their retention times, UV/visible spectral characteristics, and/or mass spectra and compared with data obtained for standard compounds tested under the same conditions. Since some carotenoids cannot be identified from their absorbance or spectrum alone, mass spectrometry (MS), MS/MS, electrospray ionization (ESI), time-of-flight (TOF) MS, etc., are often used [11,37,42]. 

The great diversity of carotenoid structures and their susceptibility to degradation make their identification and quantification very difficult. In addition, there is no reference method for their extraction and detection, they are often present at low concentrations and surrounded by numerous interfering substances, and commercial standards are often not available, further complicating their identification and quantification.

## 3. Algal Carotenoids

The carotenoid profile of algae is similar to that of higher plants in terms of their species, location, and distribution [1]. However, carotenoid composition varies qualitatively and quantitatively among different species, especially since the extraction method is not generally standardized. Various abiotic and biotic factors such as the growth stage, harvest location and period, depth, nutrient quantity and quality, temperature, salinity, light exposure, etc. also vary. The most important carotenoids from microalgae and macroalgae are astaxanthin, fucoxanthin, β-carotene, lutein, siphonaxanthin, zeaxanthin, violaxanthin, neoxanthin, and antheraxanthin [4,5,43,44].

**Figure 2:**
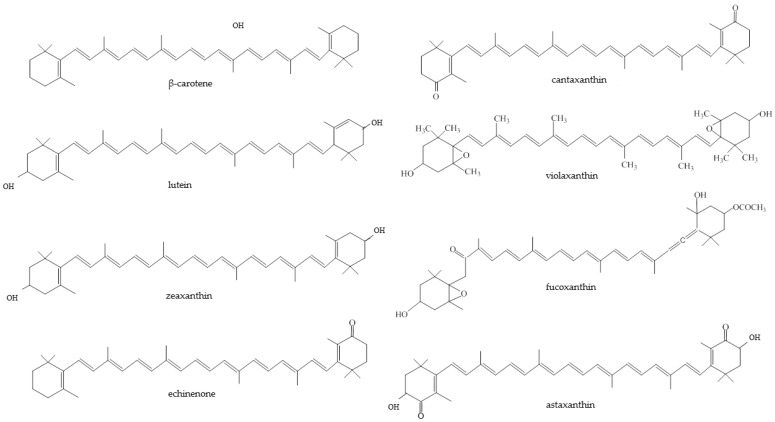
Major algal carotenoids.

Figure 2 shows the chemical structures of selected carotenoids that have been detected in algae.

### 3.1. Carotenoids in Microalgae

The main carotenoids produced by marine microalgae are β-carotene, astaxanthin, lutein, fucoxanthin, zeaxanthin, echinenone, and violaxanthin [45], but various environmental factors such as temperature, light, salinity, and nutrient content affect their production [46]. The cell walls of microalgae are an obstacle to the extraction of carotenoids. Therefore, to increase extraction yield, cell disruption (by mechanical or nonmechanical methods) is required, which is often combined with other methods to weaken and/or break the cell walls. Various nonconventional extraction techniques are also commonly used for the extraction of carotenoids from microalgal samples, e.g., supercritical fluid extraction [47], ultrasound-assisted extraction [21,26,27,28,48,49], microwave-assisted extraction [21], pulsed electric field [50], pressurized fluid extraction [22,51], magnetic-field-assisted extraction [21], etc.

Table 1 provides an overview of the studies on the extraction of carotenoids from microalgae. As can be seen, various microalgae species have been studied, and the presence of different carotenoids has been reported, such as fucoxanthin, lutein, neoxanthin, violaxanthin, α-carotene, β-carotene, etc.

Astaxanthin is an orange-red-colored xanthophyll with hydroxyl- and keto- groups [52,53,54]. Among algal species, *Haematococcus pluvialis* has the highest ability to biosynthesize astaxanthin and the greatest potential for its accumulation [5], while the microalga *Dunaliella salina* has been recognized as an important industrial source of β-carotene, a carotene with two beta rings at both ends of the molecule (β-ionone ring substitutions), especially due to its wide geographic distribution, ease of cultivation, and ability to exist under extreme environmental conditions. Its major advantage is that it serves as a precursor of vitamin A [5,46,55]. As for the solvents used, Table 1 shows that alcoholic solvents and acetone are the most commonly used.

**Table 1:** Overview of the studies on microalgal carotenoids.

Species	Origin	Solvent	Extraction Method	Extraction Conditions	Detection Method	Result	Reference
*Amphora* sp.	-	ACE	PLE	Pressure (1500 and 2000 psi); heating time (5 min); flushing solvent volume (6.6 mL); nitrogen purging (1 min)	UV/Vis, HPLC-DAD	Fucoxanthin (1.21 mg/g)	[51]
*Chaetoceros muelleri*	North Pacific	ACE	PLE	Pressure (1500 and 2000 psi); heating time (5 min); flushing solvent volume (6.6 mL); nitrogen purging (1 min)	UV/Vis, HPLC-DAD	Fucoxanthin (2.92 mg/g)	[51]
*Chlorella salina*	India	MetOH	UAME	Sonication (35 kHz, 30 min, 40 °C)	HPLC-DAD	Lutein (2.92 mg/g)	[26]
*Chlorella vulgaris*	Czech Republic	Hep-EtOH-water, THF, DCM	CSE, UAE	CSE: maceration (30 min); UAE: sonication (38 kHz, 47.7707 W/cm, 10, 20 and 30 min, 25 °C)	HPLC-DAD	Lutein (0.22–3.20 mg/g)	[28]
	Spain	EtOH	PEF	PEF pretreatment: temperature (10, 25 and 40 °C), distance between electrodes (0.25 cm), area (1.76 cm); maceration (dark); centrifugation	HPLC-DAD	Lutein (0.75 mg/g)	[50]
*Chlorococcum humicola*	Thailand	Liquefied DME, MetOH, ACE	Liquefied DME extraction, CSE	DME: time (6–60 min); temperature (30–47 °C); CSE: magnetic stirring (400 rpm); time (6–60 min); temperature (30–47 °C)	UV/Vis, HPLC-DAD	Total carotenoids (4.14 mg/g)	[56]
	India	EtOH	CSE		HPLC-UV/Vis	Violaxanthin (24.98 mg/g fw), astaxanthin (37.31 mg/g fw), lutein (48.36 mg/g fw), zeaxanthin (22.98 mg/g), α-carotene (32.90 mg/g), β-carotene (46.31 mg/g)	[57]
*Chrysotila carterae*	USA	ACE	PLE	Pressure (1500 and 2000 psi); heating time (5 min); flushing solvent volume (6.6 mL); nitrogen purging (1 min)	UV/Vis, HPLC-DAD	Fucoxanthin (1.04 mg/g)	[51]
*Desmodesmus* sp. F51	Taiwan	-	Pressure application	High-pressure homogenization (10–40 kpsi, cycles 1 to 4)	UV/Vis, HPLC-UV/Vis	Total carotenoids (0.6–8.2 mg/g), neoxanthin, violaxanthin, lutein, α-carotene, β-carotene	[58]
*Dunaliella salina*	-	CO_2_ with EtOH/MetOH	SFE	Pressure (20 and 30 MPa); temperature (308.15, 318.15, and 328.15 K); co-solvent (5%)	UV/Vis	Total carotenoids (4–25 mg/g)	[47]
	India	ACE	UAE	Vortexing (15 s); sonication (10 min); homogenization with solvent (4 days)	UV/Vis	Total carotenoids (3.2–13.9 µg/mL)	[48]
*Haematococcus pluvialis*	Canada	ACE, MetOH, EtOH	UAE	Sonication (35% amplitude at 20 kHz, 5, 15, 25, and 35 min)	UV/Vis	Total astaxanthin (0.15–0.36 mg/g)	[27]
	Czech Republic	EtOH	CSE	Vortexing (5 min) with glass beads; centrifugation (3000 rpm, 3 min)	HPLC-DAD, LC-QTOF-MS	Lutein (1.12 mg/g), β-carotene (0.89 mg/g), adonixanthin (0.17 mg/g), antheraxanthin (0.04 mg/g), neoxanthin (0.44 mg/g), astaxanthin (0.06 mg/g), echinenone (0.06 mg/g), total carotenoids (3.62 mg/g)	[59]
	Italy	ACE, EtOH, Hex, Chl/MetOH	PLE	Pressure (50 and 100 bar); temperature (20–100 °C); heating time (5 min); time (20 min); flushing solvent volume (6.6 mL); nitrogen purging (1 min)	HPLC-DAD	Astaxanthin (3.96–30.02 µg/g)	[22]
	China	EtOAc	CSE, UAE, MAE, MFAE	CSE: maceration with stirring (230 rpm, 50 min, 50 °C); UAE: sonication (100 W, 60 min, 40 °C); MAE: (30 min; 45 °C); MFAE: (field 20 mT; 50 MHz; 60 min, RT)	HPLC-DAD	Astaxanthin (76.5–111.2 mg/g)	[21]
	Brazil	DCM	Enzymatic lysis (β-1,3-glucanase, xylanes, and protease)	Enzymatic lysis in combination with ultrasonication (40 kHz)	UV/Vis	Total carotenoids (0.50–1.25 mg/g)	[23]
*Isochrysis galbana*	-		SFE	Pressure (30 MPa); temperature (50 °C), co-solvent (4% ethanol); CO_2_ flow rate (7.2 g/min for 120 min)	UV/Vis	Fucoxanthin (7.5 mg/g)	[60]
*Navicula* sp.	-	ACE	PLE	Pressure (1500 and 2000 psi); heating time (5 min); flushing solvent volume (6.6 mL); nitrogen purging (1 min)	UV/Vis, HPLC-DAD	Fucoxanthin (1.49 mg/g)	[51]
*Nanofrustulum shiloi*	Turkey	EtOH	UAE	Ultrasonic bath, 50 °C, 15 min	HPLC-DAD	Fucoxanthin (19.75–38.06 mg/g)	[61]
*Pheodactylum tricornutum*	United Kingdom	ACE	PLE	Pressure (1500 and 2000 psi); heating time (5 min); flushing solvent volume (6.6 mL); nitrogen purging (1 min)	UV/Vis, HPLC-DAD	Fucoxanthin (1.87 mg/g)	[51]
*Tisochrysis lutea*	Tahiti	ACE	PLE	Pressure (1500 and 2000 psi); heating time (5 min); flushing solvent volume (6.6 mL); nitrogen purging (1 min)	UV/Vis, HPLC-DAD	Fucoxanthin (2.05 mg/g)	[51]

UV/Vis—ultraviolet-visible; HPLC—high-performance liquid chromatography; LC—liquid chromatography; DAD—diode array detector; MS—mass spectrometry; QTOF—quadrupole time-of-flight; MetOH—methanol; ACE—acetone; EtOH—ethanol; Chl—chloroform; DCM—dichloromethane; DME—dimethyl ether; EtOAc—ethyl acetate; Hex—hexane; Hep—heptane; SFE—supercritical fluid extraction; CSE—conventional solvent extraction; PLE—pressurized liquid extraction; UAME—ultrasound-assisted microextraction; UAE—ultrasound-assisted extraction; PEF—pulsed electric field; MAE—microwave-assisted extraction; MFAE—magnetic-field-assisted extraction; RT—room temperature; fw—fresh weight.

From the reported results, the content of total carotenoids varied in the different studies and the highest content was found in *D. salina* (up to 25 mg/g) in the study by Tirado and Calvo [47]. The authors used supercritical fluid extraction as a green method for the extraction of carotenoids, while supercritical CO_2_ with EtOH/MetOH was used as the extraction solvent. Zhao et al. [21] reported an extremely high concentration of astaxanthin in *H. pluvialis* (111.2 mg/g), Ishika et al. [51] reported a similarly high concentration of fucoxanthin in *Chaetoceros muelleri* (2.92 mg/g), and Gayathri et al. [26] reported similar results for lutein in *Chlorella salina* (2.76 mg/g). Other studies also reported the presence of other carotenoids in microalgae samples: β-carotene, adonixanthin, antheraxanthin, neoxanthin, and echinenone in *H. pluvialis* [59] and neoxanthin, violaxanthin, α-carotene, and β-carotene in *Desmodesmus* sp. F51 [58].

### 3.2. Carotenoids in Green Algae (Chlorophyta)

The most abundant carotenoids in green algae are β-carotene, lutein, violaxanthin, and zeaxanthin, which are more widely distributed in green algal species than in higher plants [1]. As can be seen from the studies reported in Table 2, conventional extraction methods are generally used for the isolation and extraction of carotenoids from green algae, while only two studies [62,63] used extraction methods with ultrasound. Again, acetone is the most commonly used extraction solvent.

In the case of green algae, there are also large differences between reported results among studies, especially among studied species. Compared with microalgae, the total content of carotenoids is significantly lower (results are often reported in µg/g). The highest concentration was determined by Ak and Turker [64] in *Ulva rigida* (0.41 mg/g). Among the individual carotenoids detected by HPLC, neoxanthin was detected in *Bryopsis* sp., *Caulerpa sertularioides*, *Chaetomorpha antennina*, *Ulva fasciata*, *Ulva lactuca*, and *Ulva prolifera* [65,66,67] with the highest amount of 8.84 µg/g detected in *U. prolifera* in the study of Bhat et al. [65]; violaxanthin in *Bryopsis* sp., *Caulerpa lentillifera*, *C. sertularioides*, *C. antennina*, *Rhizoclonium riparium*, *U. lactuca*, and *U. prolifera* [65,66,67,68] with the highest amount of 8.93 µg/g detected in *C. lentillifera* in the study by Othman et al. [68]; zeaxanthin in *Bryopsis* sp., *C. lentillifera*, *Cladophora* sp., *C. antennina*, *U. fasciata*, *U. lactuca*, and *U. prolifera* [65,66,68,69] with the highest amount of 50.20 µg/g detected in *Cladophora* sp. in the study by Bhat et al. [65]; lutein in *Bryopsis* sp., *C. lentillifera*, *C. sertularioides*, *Cladophora* sp., *C. antennina*, *Monostroma nitidum*, *R. riparium*, *U. fasciata*, *U. lactuca*, *U. rigida*, and *U. prolifera* [65,66,67,68,70,71] with the highest amount of 0.30 mg/g detected in *Monostroma nitidum* in the study by Kanda et al. [70]; and β-carotene in *C. lentillifera*, *Caulerpa racemosa*, *C. sertularioides*, *Chaetomorpha linum*, *C. antennina*, *M. nitidum*, *R. riparium*, and *U. fasciata* [63,65,66,67,68,69,70,71,72] with the highest amount of 17.26 mg/g detected in *C. racemose* in the study by Magdugo et al. [63]. Among other compounds, the presence of fucoxanthin [65], neoxanthin [65,66], astaxanthin [66,67,69,72], canathaxanthin [69], β-cryptoxanthin [69], siphoxathin [66], siphonein [66], and siphonaxanthin [73] were also reported.

**Table 2:** Overview of the studies on green algae carotenoids.

Species	Origin	Solvent	Extraction Method	Extraction Conditions	Detection Method	Result	Reference
*Bryopsis* sp.	India	ACE	CSE	Maceration	HPLC-DAD	Fucoxanthin (3.44 µg/g), neoxanthin (2.11 µg/g), violaxanthin (0.84 µg/g), lutein (4.06 µg/g), zeaxanthin (1.62 µg/g)	[65]
*Caulerpa lentillifera*	Indonesia	-	-	-	UV/Vis	Total carotenoids (1.31–2.29 µg/g)	[74]
	Malaysia	ACE, Hex	CSE	Mixing; centrifugation	UV/Vis, HPLC-DAD	Total carotenoids (63.5 µg/g), zeaxanthin (21.30 µg/g), lutein (21.13 µg/g), β-carotene (10.7 µg/g), violaxanthin (8.93 µg/g)	[68]
	Malaysia	EtOH	CSE	Stirring (24 h, RT)	HPTLC, UHPLC-ESI/HRMS/MS	β-carotene (0.19 mg/g), astaxanthin (0.03 mg/g), canathaxanthin (0.15 mg/g), β-cryptoxanthin (0.013 mg/g), zeaxanthin (0.036 mg/g)	[69]
*Caulerpa racemose*	India	ACE	CSE	Homogenization (24 h, dark, RT); centrifugation (5000 rpm, 15 min)	UV/Vis	Total carotenoids (0.04 mg/g)	[75]
	Philippines	ACE	UAE	Sonication (pulse 2, amplitude 100, 2 min), maceration (dark, 4 °C, 24 h); centrifugation (36,000× *g*, 4 min)	HPLC-UV/Vis	β-carotene (17.26 mg/g)	[63]
	Indonesia	EtOH	CSE	Maceration (24 h, dark, RT, with stirring), sonication (40 °C, 30 min), filtration	UHPLC-ESI/HRMS/MS	β-carotene (0.06–0.21 mg/g), β-cryptoxanthin (0.02–0.07 mg/g), fucoxanthin (0.01–0.06 mg/g), astaxanthin (0.03–0.08 mg/g), canthaxanthin (0.04–0.16 mg/g), zeaxanthin (0.05–0.09 mg/g), lutein (0.02–0.06 mg/g)	[76]
*Cladophora rivularis*	Poland	EtOH-water	Soxhlet, UAE, MAE, SFE	Soxhlet; sonication using ultrasonic bath; MAE (800 W, yield 100%); SFE (CO_2_, flow 10 mL/min; co-solvent: ethanol, flow 1 mL/min, dynamic mode (25 min), static mode (10 min), dynamic mode (25 min), pressure 350 bar)	UV/Vis	Total carotenoids: Soxhlet (0.9 µg/mL), UAE (0.6 µg/mL), MAE (1.0 µg/mL), SFE (0.3 µg/mL)	[77]
*Caulerpa scalpelliformis*	India	ACE	CSE	Homogenization (24 h, dark, RT); centrifugation (5000 rpm, 15 min)	UV/Vis	Total carotenoids (≈0.028 mg/g)	[75]
*Caulerpa sertularioides*	Mexico	ACE	CSE	Maceration; incubation (4 °C, 24 h); centrifugation (3200× *g*, 10 min, 4 °C)	HPLC-DAD	Siphoxanthin (3.64% fw), neoxanthin (3.66% fw), violaxanthin (8.05% fw), lutein (2.38% fw), siphonein (5.8% fw), α-carotene (1.13% fw), β-carotene (5.58% fw)	[66]
*Cladophora glomerata*	Lithuanian	EtOH, ACE	CSE	Shaking; centrifugation	UV/Vis	Total carotenoids (0.17–0.23 mg/g), lutein (0.11–0.17 mg/g)	[78]
	Poland	EtOH-Water	Soxhlet, UAE, MAE, SFE	Soxhlet; sonication using ultrasonic bath; MAE (800 W, yield 100%); SFE (CO_2_, flow 10 mL/min; co-solvent: ethanol, flow 1 mL/min, dynamic mode (25 min), static mode (10 min), dynamic mode (25 min), pressure 350 bar)	UV/Vis	Total carotenoids: Soxhlet: 1.7 µg/mL; UAE: 0.5 µg/mL, MAE: 3.0 µg/mL, SFE: 1.0 µg/mL	[77]
*Cladophora* sp.	India	ACE	CSE	Maceration	HPLC-DAD	Lutein (248.67 µg/g), zeaxanthin (50.20 µg/g)	[65]
*Chaetomorpha antennina*	India	ACE	CSE	Homogenization (24 h, dark, RT); centrifugation (5000 rpm, 15 min)	UV/Vis	Total carotenoids (≈0.027 mg/g)	[75]
	India	ACE	CSE	Maceration	HPLC-DAD	Neoxanthin (2.11 µg/g), violaxanthin (0.84 µg/g), lutein (4.06 µg/g), zeaxanthin (1.62 µg/g)	[65]
*Chaetomorpha linum*	Bulgaria	MetOH, Hex-DCM	CSE	Homogenization (3 min)	HPLC-UV/FLD	Astaxanthin (0.15 µg/g), β-carotene (0.17 µg/g)	[72]
*Coelastrella* sp.	Japan	Et2O-Chl-MetOH	CSE	Homogenization by glass beads; centrifugation	HPLC	Astaxanthin (31.5%), β-carotene (0.25%)	[79]
*Codium cylindricum*	Japan	Hex-ACE	CSE	Maceration with stirring (overnight, 4 °C)	HPLC-DAD	Siphonaxanthin (68% of the total lipid fraction)	[73]
*Codium fragile*	Indonesia		-	-	UV/Vis	Total carotenoids (4.28–6.05%)	[80]
*Enteromorpha intestinalis (Linnaeus) Nees*	Turkey	ACE	-	-	UV/Vis	Total carotenoids (4.21 µg/g)	[81]
*Enteromorpha intestinalis*	Turkey	Water	CSE	Boiling (1 h)	UV/Vis	Total carotenoids (0.49 mg/g)	[64]
*Halimeda opuntia (Linnaeus) Lamouroux*	Malaysia	Chl-MetOH	CSE	Mixing (15 min); filtering; centrifugation (2000 rpm, 8 min)	UV/Vis	Total carotenoids (0.12 mg/g)	[82]
*Monostroma nitidum*	Japan	DME	-	Liquified DME extraction (vapor pressure 0.79 ± 0.02 MPa, flow 10 ± 1 mL/min, 33 min, 35 ± 1 °C)	HPLC	Lutein (0.30 mg/g)	[70]
*Rhizoclonium riparium*	Mexico	ACE	CSE	Maceration; incubation (4 °C, 24 h); centrifugation (3200× *g*, 10 min, 4 °C)	HPLC-DAD	Violaxanthin (6.12% fw), lutein (15.62% fw), dehydrolutein (2.50% fw), astaxanthin (1.52% fw), α-carotene (1.22% fw), β-carotene (1.86% fw)	[66]
*Scenedesmus* sp.	Turkey	EtOH-water (3:1 v/v), EtOAc, Hex, water	UAE	Sonication (20 min), stirring (1 h, RT), centrifugation (3800× *g*, 10 min)	UV/Vis	Total carotenoids (0.02–0.80 mg/g)	[83]
*Trentepohlia abietina*	India	ACE with BHT	CSE	Homogenization using mortar (dark); centrifugation; filtration	HPLC-UV/Vis	β-cryptoxanthin (0.15–0.34 µg/g), lutein (0.003–0.006 µg/g), β-carotene (230–585 µg/g)	[84]
*Trentepohlia arborum*	India	ACE with BHT	CSE	Homogenization using mortar (dark); centrifugation; filtration	HPLC-UV/Vis	β-cryptoxanthin (0.006–0.625 µg/g), lutein (0.002–0.006 µg/g), β-carotene (291–606 µg/g)	[84]
*Trentepohlia diffracta*	India	ACE with BHT	CSE	Homogenization using mortar (dark); centrifugation; filtration	HPLC-UV/Vis	β-cryptoxanthin (0.47–2.31 µg/g), lutein (0.001–0.005 µg/g), β-carotene (331–974 µg/g)	[84]
*Trentepohlia umbrina*	India	ACE with BHT	CSE	Homogenization using mortar (dark); centrifugation; filtration	HPLC-UV/Vis	β-cryptoxanthin (0.007–0.069 µg/g), lutein (0.001–0.003 µg/g), β-carotene (149–520 µg/g)	[84]
*Ulva fasciata*	Sri Lanka	ACE	CSE	Homogenization using mortar; centrifugation (3000 rpm, 15 min)	UV/Vis	Total carotenoids (0.17 µg/g)	[85]
	Philippines	ACE	UAE	Sonication (pulse 2, amplitude 100, 2 min), left in the dark (4 °C, 24 h); centrifugation (36000× *g*, 4 min)	HPLC-UV/Vis	β-carotene (0.72 mg/g)	[63]
	India	ACE	CSE	Maceration	HPLC-DAD	Neoxanthin (0.26 µg/g), lutein (0.90 µg/g), zeaxanthin (0.25 µg/g)	[65]
*Ulva flexuosa*	Poland	EtOH-water	Soxhlet, UAE, MAE, SFE	Soxhlet; sonication using ultrasonic bath; MAE (800 W, yield 100%); SFE (CO_2_, flow 10 mL/min; co-solvent: ethanol, flow 1 mL/min, dynamic mode (25 min), static mode (10 min), dynamic mode (25 min), pressure 350 bar)	UV/Vis	Total carotenoids: Soxhlet (1.3 µg/mL), UAE: 2.2 µg/mL, MAE (2.1 µg/mL), SFE (0.9 µg/mL)	[77]
*Ulva lactuca*	India	ACE	CSE	Homogenization (24 h, dark, RT); centrifugation (5000 rpm, 15 min)	UV/Vis	Total carotenoids (≈0.024 mg/g)	[75]
	Portugal	MetOH	UAE	Stirring (1 h); sonication; centrifugation (2935× *g*, 10 min)	UV/Vis	Total carotenoids (0.20 mg/g)	[62]
	Portugal	Water, EtOAc, EtOH	CSE	Magnetic stirring (12 h, 25 °C); centrifugation (4000 rpm, 15 min)	UV/Vis, HPLC-DAD	Total carotenoids (≈0.14–0.32 mg/g), neoxanthin, violaxanthin, astaxanthin, lutein	[67]
	Sri Lanka	ACE	CSE	Homogenization using mortar; centrifugation (3000 rpm, 15 min)	UV/Vis	Total carotenoids (0.17 µg/g)	[85]
	India	ACE	CSE	Maceration	HPLC-DAD	Neoxanthin (0.47–0.61 µg/g), violaxanthin (0.02–0.03 µg/g), lutein (21.13–23.54 µg/g), zeaxanthin (11.26–12.14 µg/g)	[65]
*Ulva ohnoi*	Brazil	Liquid N_2_, MetOH	CSE	Maceration; incubation (1 h, dark); centrifugation (12000× *g*, 10 min)	UV/Vis	Total carotenoids (18.90–32.20 µg/g)	[86]
*Ulva rigida*	Turkey	Water	CSE	Boiling (1 h)	UV/Vis	Total carotenoids (0.41 mg/g)	[64]
	Portugal	ACE	CSE	Maceration (24 h)	UHPLC-DAD-ESI-MS	Lutein (0.42–1.20 µg/mg)	[71]
*Ulva prolifera*	China	ACE	CSE	Maceration (4 °C, 12 h); centrifugation (10,000 rpm, 20 min)	UV/Vis	Total carotenoids (2.80–4.72 µg/mL)	[87]
	India	ACE	CSE	Maceration	HPLC-DAD	Fucoxanthin (0.69 µg/g), neoxanthin (8.84 µg/g), violaxanthin (3.66 µg/g), lutein (10.23 µg/g), zeaxanthin (9.47 µg/g)	[65]
	China	EtOH, PET	CSE	Maceration (60 °C, 3 min)	HPLC	Lutein (4.84–5.91 µg/g), β-carotene (0.26–1.10 µg/g)	[88]
*Valoniopsis pachynema*	India	ACE	CSE	Homogenization (24 h, dark, RT); centrifugation (5000 rpm, 15 min)	UV/Vis	Total carotenoids (≈0.022 mg/g)	[75]

≈—approximately (data from graphical presentation of the results); UV/Vis—ultraviolet-visible; HPLC—high-performance liquid chromatography; UHPLC—ultra-high-performance liquid chromatography; DAD—diode array detector; FLD—fluorescence detector; HPTLC—high-performance thin layer chromatography; HRMS—high- resolution mass spectrometry; ESI—electrospray ionization; MS—mass spectrometry; MetOH—methanol; ACE—acetone; EtOH—ethanol; Chl—chloroform; DCM—dichloromethane; DME—dimethyl ether; Et2O—diethyl ether; EtOAc—ethyl acetate; Hex—hexane; PET—petroleum ether; CSE—conventional solvent extraction; UAE—ultrasound-assisted extraction; BHT—butylated hydroxyl toluene; RT—room temperature; fw—fresh weight.

### 3.3. Carotenoids in Brown Algae (Phaeophyta)

Brown algae contain more xanthophylls than carotenes, and this prevalence is responsible for their coloration and activity [89]. The major pigments in brown algae are fucoxanthin, β-carotene, and violaxanthin [1]. Fucoxanthin is the major carotenoid in brown algae. It is an allelic carotenoid, a 5,6-monoepoxide that has nine conjugated double bonds and oxygen-containing functional groups (hydroxyl, epoxy, carbonyl, and carboxyl groups). This unique structure distinguishes it from other carotenoids [5,90,91]. Its content varies greatly depending on location, season, and other factors (pH and salinity). For example, fucoxanthin concentration has been found to increase in winter in response to sunlight limitation [90,91].

Fucoxanthin and the other carotenoids were isolated from different species of brown algae using different extraction and detection methods that resulted in different concentrations. Again, the most commonly used method to isolate carotenoids was CSE, followed by UAE [62,92,93,94], but the use of other methods has also been reported [94,95,96]. In addition, hexane and alcoholic solvents were used in most cases (Table 3).

The total carotenoid content in the reported studies ranged from 57 µg/g in *Padina pavonica* [68] to 406 mg/g in *Dictyota dentata* [97]. The highest fucoxanthin content (27.40 mg/g) was detected in *Sargassum polycystum* [69]. Other carotenoids were detected at lower concentrations in brown algae. Tabakaeva and Tabakaev [98] analyzed the carotenoid composition of *Sargassum miyabei*, which was as follows: fucoxanthin (57.9%), zeaxanthin (12.5%), violaxanthin (4.9%), neoxanthin (3.1%), β-carotene (2.4%), and α-carotene (0.2%). Garcia-Perez et al. [89] detected seven carotenoids in nine brown algae; six xanthophylls (fucoxanthin, violaxanthin, auroxanthin, dihydrolutein, zeaxanathin, and fucoxanthin derivative) and one carotene (β-carotene). Lourenço-Lopes et al. [92] reported the content of fucoxanthin (between 0.67 and 9.54 mg/g) and β-carotene (between 0.01 and 0.30 mg/g) in nine brown algae, namely, *Ascophyllum nodosum*, *Bifurcaria bifurcate*, *Fucus spiralis*, *Himanthalia elongate*, *Laminaria ochroleuca*, *Laminaria saccharina*, *Pelvetia canaliculata*, *Sargassum muticum*, and *Undaria pinnatifida*. Astaxantin was found in *Cystoseira barbata* and *Cystoseira crinita* in the study by Dobreva et al. [72], *D. dentata* and *Padina durvillaei* in the study by Osuna-Ruiz et al. [66], and *Sargassum polycystum* in the study by Balasubramaniam et al. [69]. Among other carotenoids, lutein was found in *Fucus vesiculosus* [71], *S. polycystum* [69], and *P. pavonica* [68]; violaxanthin and 19-Hex-fucoxanthin were found in *P. durvillaei* [66]; zeaxanthin was found in *P. pavonica* [68] and *S. polycystum* [69]; and canathaxanthin and cryptoxanthin were found in *S. polycystum* [69].

**Table 3:** Overview of the studies on brown algae carotenoids.

Species	Origin	Solvent	Extraction Method	Extraction Conditions	Detection Method	Result	Reference
*Ascophyllum nodosum*	Spain	EtOH	UAE	Vortexing (30 s); sonication (500 W, 55 min); centrifugation (8400 rpm, RT, 7 min)	HPLC-DAD	Fucoxanthin (2 mg/g), β-carotene (0.05 mg/g), other carotenoids (1.2 mg of fucoxanthin equivalents/g)	[92]
*Bifurcaria bifurcata*	Spain	EtOH	UAE	Vortexing (30 s); sonication (500 W, 55 min); centrifugation (8400 rpm, RT, 7 min)	HPLC-DAD	Fucoxanthin (0.71 mg/g), β-carotene (0.08 mg/g), other carotenoids (1.52 mg of fucoxanthin equivalents/g)	[92]
*Cystoseira barbata*	Turkey	Water	CSE	Boiling (1 h)	UV/Vis	Total carotenoids (2.195 mg/g)	[64]
	Bulgaria	MetOH, Hex-DCM	CSE	Homogenization (3 min); saponification (50 °C, 30 min); extracted twice in *n*-hexane	HPLC-UV/FLD	Astaxanthin (3.0 µg/g fw), β-carotene (55.7 µg/g fw)	[72]
*Cystoseira crinita*	Bulgaria	MetOH, Hex-DCM	CSE	Homogenization (3 min); saponification (50 °C, 30 min); extracted twice in *n*-hexane	HPLC-UV/FLD	Astaxanthin (1.39 µg/g fw), β-carotene (18.8 µg/g fw)	[72]
*Dictyota dentata*	Indonesia	ACE-MetOH	CSE	Homogenization using mortar; vortexing; centrifugation; drying with N_2_	UV/Vis, HPLC-DAD	Total carotenoids (4.06 mg/g), fucoxanthin (4.11 mg/g, 0.29 mg/g fw) β-carotene (0.78 mg/g, 0.08 mg/g fw)	[97]
*Fucus spiralis*	Spain	EtOH	UAE	Vortexing (30 s); UAE: sonication (500 W, 55 min); centrifugation (8400 rpm, RT, 7 min)	HPLC-DAD	Fucoxanthin (2.48 mg/g), β-carotene (0.08 mg/g), other carotenoids (1.41 mg of fucoxanthin equivalents/g)	[92]
*Fucus vesiculosus*	Portugal	ACE	CSE	Maceration (24 h)	UHPLC-DAD-ESI-MS	Lutein (0.03–0.18 µg/mg), β-carotene (0.24–0.55 µg/mg), fucoxanthin (0.78–1.79 µg/mg)	[71]
*Himanthalia elongata*	Ireland	Hex, Et_2_O, Chl	CSE	Maceration, filtration, centrifugation (9168× *g*, 15 min)	LC-ESI-MS	Fucoxanthin (18.6 mg/g)	[99]
	Spain	EtOH	UAE	Vortexing (30 s); sonication (500 W, 55 min); centrifugation (8400 rpm, RT, 7 min)	HPLC-DAD	Fucoxanthin (0.67 mg/g), β-carotene (0.01 mg/g), other carotenoids (0.33 mg of fucoxanthin equivalents/g)	[92]
*Laminaria ochroleuca*	Spain	EtOH	UAE	Vortexing (30 s); sonication (500 W, 55 min); centrifugation (8400 rpm, RT, 7 min)	HPLC-DAD	Fucoxanthin (4.35 mg/g), β-carotene (0.03 mg/g), other carotenoids (0.48 mg of fucoxanthin equivalents/g)	[92]
*Laminaria saccharina*	Spain	EtOH	UAE	Vortexing (30 s); sonication (500 W, 55 min); centrifugation (8400 rpm, RT, 7 min)	HPLC-DAD	Fucoxanthin (9.54 mg/g), β-carotene (0.07 mg/g), other carotenoids (0.48 mg of fucoxanthin equivalents/g)	[92]
*Padina australis*	Indonesia	ACE-MetOH	CSE	Homogenization using mortar; vortexing; centrifugation; drying with N_2_	UV/Vis, HPLC-DAD	Total carotenoids (3.56 mg/g), fucoxanthin (1.64 mg/g, 0.22 mg/g fw), β-carotene (0.35 mg/g, 0.08 mg/g fw)	[97]
*Padina durvillaei*	Mexico	ACE	CSE	Homogenization; incubation (24 h, 4 °C); centrifugation (3200× *g*, 10 min, 4 °C)	HPLC-DAD	Fucoxanthin (33.9% fw), violaxanthin (4.5% fw), 19-Hex-fucoxanthin (5.80% fw), astaxanthin (0.42% fw), β-carotene (4.16% fw)	[66]
*Padina pavonica*	Malaysia	ACE, Chl	CSE	Saponification; mixing; centrifugation	UV/Vis, HPLC-DAD	Total carotenoids (0.1 mg/g), zeaxanthin (10.87 µg/g), lutein (7.21 µg/g), β-carotene (9.14 µg/g)	[68]
*Pelvetia canaliculata*	Spain	EtOH	UAE	Vortexing (30 s); sonication (500 W, 55 min); centrifugation (8400 rpm, RT, 7 min)	HPLC-DAD	Fucoxanthin (2.07 mg/g), β-carotene (0.06 mg/g), other carotenoids (0.45 mg of fucoxanthin equivalents/g)	[92]
*Saccharina japonica*	South Korea	Supercritical CO_2_	SFE	Temperature (40–50 °C), pressure (200–300 bar), mixing ratio (27–75%)	HPLC	Fucoxanthin (2.08 mg/g)	[95]
*Sargassum crassifolium*	Indonesia	ACE-MetOH		Homogenization using mortar; vortexing; centrifugation; drying with N_2_	UV/Vis, HPLC-DAD	Total carotenoids (1.01 mg/g), fucoxanthin (0.75 mg/g), fucoxanthin (0.09 mg/g fw), β-carotene (0.31 mg/g fw, 0.07 mg/g)	[97]
*Sargassum fusiforme*	China	Ethyl lactate	UAE	Maceration (2 h, dark); sonication (500 W, 20 kHz), centrifugation (9000× *g*, 10 min, 4 °C)	HPLC	Fucoxanthin (0.6 mg/g by ethyl lactate)	[93]
*Sargassum miyabei*		Me_2_CO, Hex	CSE	Homogenization using mortar; filtration; separation over Al_2_O_3_	HPLC-DAD	In the thallus (fucoxanthin: 57.9%, zeaxanthin: 12.5%, violaxanthin: 4.9%, neoxanthin: 3.1%, β-carotene: 2.4%, α-carotene: 0.2%) In the phylloids (fucoxanthin: 63.2%, zeaxanthin: 10.8%, violaxanthin: 2.7%, neoxanthin: 7.7%, β-carotene: 3.6%)	[98]
*Sargassum muticum*	Spain	EtOH	UAE	Vortexing (30 s); sonication (500 W, 55 min); centrifugation (8400 rpm, RT, 7 min)	HPLC-DAD	Fucoxanthin (5.79 mg/g), β-carotene (0.06 mg/g), other carotenoids (0.94 mg of fucoxanthin equivalents/g)	[92]
*Sargassum polycystum*	Malaysia	ACE, EtOH, MetOH	CSE	Shaking (40 °C, 24 h), centrifugation (2072× *g*, 10 min); filtration, drying	HPLC	Fucoxanthin (0.28 mg/g)	[100]
	Malaysia	EtOH	CSE	Stirring (24 h, RT), filtration	HPTLC, UHPLC-ESI/HRMS/MS	Astaxanthin (0.26 mg/g), canathaxanthin (0.22 mg/g), β-cryptoxanthin 0.06 mg/g), fucoxanthin: 27.40 mg/g), zeaxanthin (0.14 mg/g), lutein (0.12 mg/g)	[69]
*Sargassum* sp.	Mexico	EtOH	UAE, shock wave-assisted extraction	UAE: sonication (40 kHz, 160 W, 30 min); shock wave-assisted extraction: (delay between waves 50 and 950 μs, wave rate 0.5 Hz, voltage 3 and 6 kV, duration 404 ± 8 ns and 196 ± 8 ns)	UV/Vis, HPLC-UV/Vis	Fucoxanthin (UV/Vis: 0.29–0.39 mg/g, HPLC: 0.29–0.41 mg/g)	[94]
*Scytosiphon lomentaria*	Turkey	Water	CSE	Boiling (1 h)	UV/Vis	Total carotenoids (0.794 mg/g)	[64]
*Sphaerotrichia divaricata*	Japan	Chl-MetOH	CSE	Precipitation from lipid fraction	HPLC-DAD	Fucoxanthin (1.15 mg/g)	[101]
*Turbinaria conoides*	Indonesia	ACE-MetOH		Homogenization using mortar; vortexing; centrifugation; drying with N_2_	UV/Vis, HPLC-DAD	Total carotenoids (0.55 mg/g), fucoxanthin (0.43 mg/g, 0.13 mg/g fw) β-carotene (0.16 mg/g, 0.07 mg/g fw)	[97]
*Undaria pinnatifida*	Japan	EtOH	SFE-CO_2_	Pressure (4000 psi), temperature (40 °C), Time (150 min); CO_2_ flow rate (1 mL/min)	HPLC-UV/Vis	Fucoxanthin (22.09 mg/g)	[96]
	Spain	EtOH	UAE	Vortexing (30 s); sonication (500 W, 55 min); centrifugation (8400 rpm, RT, 7 min)	HPLC-DAD	Fucoxanthin (6.15 mg/g), β-carotene (0.30 mg/g), other carotenoids (2.42 mg of fucoxanthin equivalents/g)	[92]
*Zonaria tournefortii*	Portugal	MetOH	UAE	Stirring (1 h); sonication; centrifugation (2935× *g*, 10 min)	UV/Vis	Total carotenoids (2.98 mg/g)	[62]

UV/Vis—ultraviolet-visible; HPLC—high-performance liquid chromatography; LC—liquid chromatography; UHPLC—ultra-high-performance liquid chromatography; DAD—diode array detector; HPTLC—high-performance thin layer chromatography; HRMS—high- resolution mass spectrometry; ESI—electrospray ionization; MS—mass spectrometry; FLD—fluorescence detector; DCM—dichloromethane; MetOH—methanol; ACE—acetone; EtOH—ethanol; Chl—chloroform; Et2O—diethyl ether; Hex—hexane;; SFE—supercritical fluid extraction; CSE—conventional solvent extraction; UAE—ultrasound-assisted extraction; RT—room temperature; fw—fresh weight.

### 3.4. Carotenoids in Red Algae (Rhodophyta)

The dominant carotenoids in red algae are zeaxanthin, lutein, and α- and β-carotene, but in contrast to the α-:β-carotene ratio typical of higher plants, the α-carotene content is much higher in red algae [1]. A review of recent studies identifying carotenoids in red algae is presented in Table 4. Red algal carotenoids were mostly extracted by conventional extraction with solvents (alcohols, acetone, hexane, ethyl acetate, or water), while only some studies used ultrasound to assist the extraction [62,63,64,65,66,67,68,69,70,71,72,73,74,75,76,77,78,79,80,81,82,83,84,85,86,87,88,89,90,91,92,93,94,95,96,97,98,99,100,101,102].

The highest reported total carotenoid content from Table 4 was reported by Hossain et al. [103] in acetone extracts of *Gelidium pusillum* from Bangladesh (52.7 mg/g). Lutein was found in *Ahnfeltia plicata*, *Ceramium sp.*, *Chondrus crispus*, *Delesseria sanguinea*, *Dilsea carnosa*, *Eucheuma denticulatum*, *Furcellaria lumbricalis*, *Furcellaria lumbricalis*, *Gracilaria changii*, *Gracilaria corticata*, *Gracilaria tikvahiae*, *Kappaphycus alvarezii*, *Odonthalia dentate*, *Palmaria palmate Pyropia yezoensis*, and *Spyridia filamentosa* [65,68,69,102,104]. In addition to lutein being present in all nine algae studied by Razi Parjikolaei et al. [104] (*A. plicata*, *C. crispus*, *D. sanguinea*, *D. carnosa*, *F. lumbricalis*, *Gracilaria vermiculophylla*, *O. dentate*, *P. palmata*, and *Phycodrys rubens*), the presence of zeaxanthin (from 0.66 to 1.7 µg/g) and β-carotene (from 2.2 to 8.8 µg/g) was also confirmed. Balasubramaniam et al. [69] also detected astaxanthin, β-cryptoxanthin, and fucoxanthin in *Eucheuma denticulatum.* The presence of astaxanthin in red algae was also reported by Dobreva et al. [72] in *Gelidium crinale*. Among others, neoxanthin and violaxanthin were found in *Grateloupia filicina* [65], while α-cryptoxanthin, β-cryptoxanthin, lutein-5,6-epoxide, and antheraxanthin were detected in *P. yezoensis* [105].

**Table 4:** Overview of the studies on red algae carotenoids.

Species	Origin	Solvent	Extraction Method	Extraction Conditions	Detection Method	Result	Reference
*Ahnfeltia plicata*	Denmark	ACN	CSE	Homogenization using mortar	HPLC-MS	Lutein (≈1.8 µg/g), zeaxanthin (≈1.7 µg/g), β-carotene (≈2.2 µg/g)	[104]
*Amphiroa rigida*	Portugal	ACE	CSE	Homogenization using mortar (10 min); stirring (30 min); centrifugation (12,500× *g*, 20 min, 4 °C)	UV/Vis	Total carotenoids (1.05 µg/g fw)	[106]
*Asparagopsis taxiformis*	Portugal	MetOH	UAE	Stirring (1 h); sonication; centrifugation (2935× *g*, 10 min)	UV/Vis	Total carotenoids (0.13 mg/g)	[62]
*Ceramium ciliatum*	Portugal	ACE	CSE	Homogenization using mortar (10 min); stirring (30 min); centrifugation (12,500× *g*, 20 min, 4 °C)	UV/Vis	Total carotenoids (0.32 µg/g fw)	[106]
*Ceramium rubrum*	Turkey	ACE		-	UV/Vis	Total carotenoids (2.14 µg/g)	[81]
*Ceramium* sp.	India	ACE	CSE	Maceration	HPLC-DAD	Fucoxanthin (4.85 µg/g), lutein (3.26 µg/g), zeaxanthin (0.66 µg/g)	[65]
*Chondrus crispus*	Portugal	ACE	CSE	Homogenization using mortar (10 min); stirring (30 min); centrifugation (12,500× *g*, 20 min, 4 °C)	UV/Vis	Total carotenoids (0.12 µg/g fw)	[106]
	Portugal	MetOH	UAE	Stirring (1 h); sonication; centrifugation (2935× *g*, 10 min)	UV/Vis	Total carotenoids (0.21 mg/g)	[62]
	Denmark	ACN	CSE	Homogenization using mortar	HPLC-MS	Lutein (≈3.0 µg/g), zeaxanthin (≈2.6 µg/g), β-carotene (≈7.8 µg/g)	[104]
*Corallina mediterranea*	Egypt	ACE	CSE	Maceration (72 h, RT, dark) with intermittent shaking	UV/Vis	Total carotenoids (≈0.02 mg/g fw)	[107]
*Delesseria sanguinea*	Denmark	ACN	CSE	Homogenization using mortar	HPLC-MS	Lutein (≈3.7 µg/g), zeaxanthin (0.9 µg/g), β-carotene (≈4.2 µg/g)	[104]
*Dilsea carnosa*	Denmark	ACN	CSE	Homogenization using mortar	HPLC-MS	Lutein (≈7.0 µg/g), β-carotene (≈3.8 µg/g)	[104]
*Ellisolandia elongata*	Portugal	ACE	CSE	Homogenization using mortar (10 min); stirring (30 min); centrifugation (12,500× *g*, 20 min, 4 °C)	UV/Vis	Total carotenoids (0.89 µg/g fw)	[106]
*Eucheuma denticulatum*	Malaysia	EtOH	CSE	Stirring (24 h, RT)	HPTLC, UHPLC-ESI/HRMS/MS	β-carotene (0.047 mg/g), astaxanthin (0.03 mg/g), β-cryptoxanthin 0.036 mg/g), zeaxanthin (0.21 mg/g), lutein (0.88 mg/g), fucoxanthin (0.04 mg/g)	[69]
	Malaysia	ACE, Hex	CSE	Mixing; centrifugation	UV/Vis, HPLC-DAD	Total carotenoids (33 µg/g), zeaxanthin (3.61 µg/g), lutein (9.57 µg/g), β-carotene (2.44 µg/g)	[68]
*Furcellaria lumbricalis*	Denmark	ACN	CSE	Homogenization using mortar	HPLC-DAD	Lutein (13 µg/g), β-carotene (≈0.008 µg/g)	[104]
*Gelidium crinale*	Bulgaria	MetOH, Hex-DCM	CSE	Homogenization (3 min)	HPLC-UV/FLD	Astaxanthin (2.0 µg/g), β-carotene (33.8 µg/g)	[72]
*Gelidium pusillum*	Bangladesh	ACE	CSE	Incubation with shaking (250 rpm, 90 min, 20 °C); centrifugation (3000 rpm, 15 min)	UV/Vis	Total carotenoids (52.7 mg/g)	[103]
*Gigartina acicularis*	Turkey	Water	CSE	Boiling (1 h)	UV/Vis	Total carotenoids (0.59 µg/g)	[64]
*Gracilaria changii*	Malaysia	Hex-ACE-EtOH	CSE	Shaking; centrifugation (3000 rpm, 5 min, 4 °C)	UV/Vis	Total carotenoids (7.34 mg β-carotene equivalent/g)	[108]
	Indonesia	ACE	CSE	Maceration (24 h, dark)	UV/Vis	Total carotenoids (0.24 µg/g)	[109]
*Gracilaria corticata*	India	ACE	CSE	Incubation (45 min, dark); centrifugation (10,000× *g*, 5 min)	UV/Vis	Total carotenoids (12.82 µg/g)	[110]
	India	ACE	CSE	Maceration	HPLC-DAD	Fucoxanthin (6.06 µg/g), lutein (0.26 µg/g), zeaxanthin (0.65 µg/g)	[65]
*Gracilaria edulis*	India	ACE	CSE	Incubation (dark, 45 min); centrifugation (10,000× *g*, 5 min)	UV/Vis	Total carotenoids (2.99 µg/g)	[110]
*Gracilaria tikvahiae*	Malaysia	ACE, Hex	CSE	Mixing; centrifugation	UV/Vis, HPLC-DAD	Total carotenoids (25.1 µg/g), zeaxanthin (4.15 µg/g), lutein (8.86 µg/g), β-carotene (3.05 µg/g)	[68]
*Gracilaria vermicu-lophylla*	Denmark	ACN	CSE	Homogenization using mortar	HPLC-DAD	Zeaxanthin (0.93 µg/g), β-carotene (≈8.8 µg/g)	[104]
	Mexico	ACE	CSE	Maceration; incubation (24 h, 4 °C); centrifugation (3200× *g*, 10 min, 4 °C)	HPLC-DAD	Zeaxanthin (7.83% fw), β-carotene (6.93% fw)	[66]
*Grateloupia filicina*	India	ACE	CSE	Maceration	HPLC-DAD	Fucoxanthin (3.45 µg/g), neoxanthin (2.04 µg/g), violaxanthin (20.65 µg/g), lutein (18.38 µg/g), zeaxanthin (2.16 µg/g)	[65]
*Grateloupia sp.*	India	ACE	CSE	Maceration	HPLC-DAD	Lutein (166.58 µg/g), zeaxanthin (36.34 µg/g)	[65]
*Halymenia durvillei*	Indonesia	Hex, ACE, EtOH	CSE	Incubation (overnight, RT, dark)	UV/Vis	Total carotenoids (2.64–28.65 µg/g)	[111]
*Hypnea musciformis*	Bangladesh	ACE	CSE	Incubation with shaking (250 rpm, 90 min, 20 °C); centrifugation (3000 rpm,15 min)	UV/Vis	Total carotenoids (31.6 mg/g)	[103]
*Jania rubens*	Egypt	ACE	CSE	Maceration (72 h, RT, dark) with intermittent shaking	UV/Vis	Total carotenoids (≈0.02 mg/g fw)	[107]
	Portugal	ACE	CSE	Homogenization using mortar (10 min); stirring (30 min); centrifugation (12,500× *g*, 20 min, 4 °C)	UV/Vis	Total carotenoids (1.88 µg/g fw)	[106]
*Kappaphycus alvarezii*	Brazil	Hex-ACE	UAE	Sonication (2.5 GHz, 1 h, dark); centrifugation (4000 rpm, 10 min)	UV/Vis, HPLC-UV/Vis	Lutein (112.02 µg/g), zeaxanthin (32.15 µg/g), α-carotene (13.36 µg/g), β-carotene (0.60 mg/g), β-carotene (0.24 mg/g)	[102]
*Kappaphycus striatum*	Malaysia	ACE, Hex	CSE	Mixing; centrifugation	UV/Vis, HPLC-DAD	Total carotenoids (57.0 µg/g), zeaxanthin (4.47 µg/g), lutein (38.60 µg/g), β-carotene (7.59 µg/g)	[68]
*Liagora viscida*	Portugal	ACE	CSE	Homogenization using mortar (10 min); stirring (30 min); centrifugation (12,500× *g*, 20 min, 4 °C)	UV/Vis	Total carotenoids (≈0.55 µg/g fw)	[106]
*Nemalion elminthoides*	Portugal	MetOH	UAE	Stirring (1 h); sonication; centrifugation (2935× *g*, 10 min)	UV/Vis	Total carotenoids (0.09 mg/g)	[62]
*Neopyropia yezoensis*	China	ACE, EtOAc, water	CSE	Vortexing (15 s); centrifugation (10,000× *g*, 5 min, 4 °C)	HPLC-DAD	Lutein (5.0 mg/g), β-carotene (0.6 mg/g), zeaxanthin (0.2 mg/g), α-carotene (0.14 mg/g)	[112]
*Mesophyllum lichenoides*	Portugal	ACE	CSE	Homogenization using mortar (10 min); stirring (30 min); centrifugation (12,500× *g*, 20 min, 4 °C)	UV/Vis	Total carotenoids (0.15 µg/g fw)	[106]
*Odonthalia dentata*	Denmark	ACN	CSE	Homogenization using mortar	HPLC-MS	Lutein (1.4 µg/g), zeaxanthin (≈4.0 µg/g), β-carotene (≈2.2 µg/g)	[104]
*Osmundea pinnatifida*	Portugal	ACE	CSE	Homogenization using mortar (10 min); stirring (30 min); centrifugation (12,500× *g*, 20 min, 4 °C)	UV/Vis	Total carotenoids (≈0.75 µg/g fw)	[106]
*Palmaria palmata*	Denmark	ACN	CSE	Homogenization using mortar	HPLC-MS	Lutein (≈1.9 µg/g), β-carotene (1.9 µg/g)	[104]
*Phycodrys rubens*	Denmark	ACN	CSE	Homogenization using mortar	HPLC-MS	Lutein (15.2 µg/g), zeaxanthin (≈3.2 µg/g), β-carotene (15.7 µg/g)	[104]
*Plocamium cartilagineum*	Portugal	ACE	CSE	Homogenization using mortar (10 min); stirring (30 min); centrifugation (12,500× *g*, 20 min, 4 °C)	UV/Vis	Total carotenoids (0.40 µg/g fw)	[106]
*Porphyra umbilicalis*	Portugal	ACE	CSE	Homogenization using mortar (10 min); stirring (30 min); centrifugation (12,500× *g*, 20 min, 4 °C)	UV/Vis	Total carotenoids (1.88 µg/g fw)	[106]
*Pterocladia capillacea*	Egypt	ACE	CSE	Maceration (72 h, RT, dark) with intermittent shaking	UV/Vis	Total carotenoids (0.092 mg/g)	[107]
*Pyropia orbicularis*	Chile	Hex-ACE-EtOH	CSE	Homogenization; centrifugation (2130× *g*, 15 min)	UV/Vis	Total carotenoids (58.6 µg/g)	[113]
*Pyropia yezoensis*	Japan	MetOH, Chl	CSE	Maceration (24 h, RT, dark)	LC-MS, 1H-NMR	Lutein (3.46 mg/g), zeaxanthin, α-carotene, β-carotene, α-cryptoxanthin, β-cryptoxanthin, lutein-5,6-epoxide, antheraxanthin	[105]
	China	MetOH-ACE	CSE	Homogenization; maceration	HPLC-DAD	Lutein, zeaxanthin, α-carotene, β-carotene	[114]
*Spyridia filamentosa*	Mexico	ACE	CSE	Maceration; incubation (24 h, 4 °C); centrifugation (3200× *g*, 10 min, 4 °C)	HPLC-DAD	Lutein (33.28% fw), dihydrolutein (3.70% fw), astaxanthin (0.43% fw), α-carotene (2.30% fw), β-carotene (9.62% fw)	[66]
*Sphaerococcus coronopifolius*	Portugal	ACE	CSE	Homogenization using mortar (10 min); stirring (30 min); centrifugation (12,500× *g*, 20 min, 4 °C)	UV/Vis	Total carotenoids (0.70 µg/g fw)	[106]
*Spyridia filamentosa*	Mexico	ACE	CSE	Homogenization using mortar	HPLC-DAD	Lutein (13 µg/g), β-carotene (≈0.008 µg/g)	[104]

≈—approximately (data from graphical presentation of the results); UV/Vis—ultraviolet-visible; HPLC—high-performance liquid chromatography; LC—liquid chromatography; UHPLC—ultra-high-performance liquid chromatography; DAD—diode array detector; HPTLC—high-performance thin layer chromatography; HRMS—high-resolution mass spectrometry; ESI—electrospray ionization; MS—mass spectrometry; FLD—fluorescence detector; NMR—nuclear magnetic resonance; DCM—dichloromethane; MetOH—methanol; ACN—acetonitrile; ACE—acetone; EtOAc—ethyl acetate; EtOH—ethanol; Chl—chloroform; Hex—hexane; CSE—conventional solvent extraction; UAE—ultrasound-assisted extraction; RT—room temperature; fw—fresh weight.

## 4. Potential Applications of Algal Carotenoids

The most commercially interesting carotenoids include astaxanthin, β-carotene, lutein, and zeaxanthin, which are widely distributed in algae, making these organisms an important source of natural carotenoids. Fucoxanthin, abundant in brown algae, is considered a therapeutic and nutritional ingredient with a unique chemical structure that enables its reactions in many physiological functions and ensures its strong biological properties. Algal carotenoids (fucoxanthin and others) derived from Indian brown algae (*Padina tetrastromatica*) have been studied against oxidative stress in rats [115]. It was found that lipid oxidation induced by retinol deficiency (plasma and liver) was reduced by the supplementation of fucoxanthin (plasma 7–85% and liver 24–72%) versus β-carotene (plasma-51–76% and liver 33–65%) by enhancing the activity of catalase and glutathione transferase enzymes. Similarly, Jang et al. [116] reported the ability of fucoxanthin from *Laminaria japonica* to impart hepatoprotective effects under oxidative stress, suggesting its inclusion in the formulation of nutraceuticals. For other algae-derived carotenoids, protective properties such as cardioprotective, hepatoprotective, photoprotective, renal protective, and various health-promoting and beneficial properties such as antioxidant, anti-obesity, antitumor, antidiabetic, anti-inflammatory, and hepatoprotective have been confirmed in the literature [29,45,91,101,115,116,117,118,119,120,121,122,123]. Due to these properties, algae-derived carotenoids have been investigated for various applications. Most commonly, they are used as dietary supplements and food colorants, for the production of functional/nutraceutical foods and animal feeds, for the formulation of food packaging, in health care, and in cosmetics [65,124,125,126]. The main challenges for their industrial application are the extraction method, reported variations in yield, and their unstable nature [124,126,127,128]. Based on the proposed concerns, several studies have been conducted and reported on the ability of carotenoid-rich algae as a blend, coating, film, and additive to various food matrices, which have been carefully presented in previous reports [124,129,130,131].

Taking carotenoids from marine algae as natural antioxidants has been shown to be effective in reducing obesity and weight gain. The molecular mechanism of obesity has been linked to inflammation and oxidative stress, which lead to the development of other metabolic diseases (e.g., type 2 diabetes, hypertension, and liver disease). Antioxidants from marine algae have been suggested as potential replacements for conventional treatments such as surgery or drugs. Carotenoids from algae have been associated with the regulation of key factors in adipogenesis, glucose levels, and fatty acid metabolism [119]. In addition, algal carotenoids can be used for the nutrient fortification of foods. They have been incorporated as powders or oils into various food matrices such as pasta, bread, cookies, vegetable soup, and yogurt [132,133,134]. The formulation and dosage for each product still need to be optimized as high doses of some carotenoids or algae are considered unacceptable by consumers, mainly because of their color and flavor properties. However, in the right concentration, they could provide nutrient fortification and antioxidant activity and even prolong the shelf life of foods. On the contrary, there are studies confirming that a pigment such as fucoxanthin can be successfully used as a colorant to improve the appearance of and provide bioactivity to foods and beverages [90].

Algal carotenoids showed potential in food packaging film formulations. Films formulated with algal extract (*Fucus vesiculosus*) exhibited lower lipid oxidation in chicken breast samples, which was attributed to higher carotene content [135]. Similarly, Sáez et al. [136] reported the preservative action of carotenoid-rich water extracts from algae on rainbow trout fillets. The application inhibited the growth of total viable counts and lipid oxidation and helped to preserve the quality of fillets by improving their water holding capacity [136]. Recently, Pereira et al. [137] summarized studies and meta-analyses on the health-related properties and effects of marine-derived carotenoids.

Algae-derived carotenoids have also been added to feed to improve the color of fish (salmon and trout), crustaceans, and eggs [138]. These organisms represent a conventional food source for humans, and diets rich in carotenoids have been associated with health benefits. The carotenoid profile is largely dependent on feed composition [139]. Traditionally, carotenoids have been used extensively as colorants in foods; however, with the development of natural sources for the extraction of carotenoids and their extensive assimilability to synthetic forms, they found further applications in the feed industry [138]. The application of algal pigment extracts rich in carotenoids has shown the ability to enhance immunity to *Vibrio* infections and promote weight gain in shrimp production [140]. Another study by Abdel-Rahim et al. [141] concluded that dietary supplementation of algae apart from the findings of Aftab Uddin et al. [140] had imparted cold tolerance. In the case of beef feed supplemented with algae, it lowered the carbon footprint by lowering the methane production in vitro without loss of efficiency, which supported its use in feeds [142].

Pigments from marine algae are also used as active ingredients in cosmetics, where their addition delays skin aging and protects against UV radiation, which leads to the formation of reactive oxygen species. The formation of these components can damage DNA and lead to hyperpigmentation, premature aging, sunburn, and skin cancer [143]. The presence of carotenoids in skin tissue occurs through two mechanisms: diffusion from the body, e.g., adipose tissue and plasma, and/or secretion by sebaceous glands and reabsorption. In addition, the content and profile of carotenoids reflect those present in plasma, the most important being lutein, β-carotene, lycopene, zeaxanthin, β-cryptoxanthin, and colorless pigments (phytoene and phytofluene). The presence of carotenoids on the skin surface is associated with the reduction of oxidation and inflammation, leading to other effects such as inhibition of metalloproteases, inhibition of UVA-induced expression of heme oxygenase 1, prevention of mitochondrial DNA mutations, and photoimmunomodulation. In a study by Grether-Beck et al. [144], oral supplementation of lutein and lycopene was shown to result in photoprotection. The effect was at the molecular level by inhibiting UVA1 and UVA/B-induced gene expression. Astaxanthin was found to decrease the expression of matrix metalloproteinases that degrade collagen and elastin. It has also been associated with improvement in sebum oil levels, wrinkling, elasticity, and hydration [145].

Brown algae have also been used as fertilizers with improved effects on plants. Nurjannah et al. [146] used a fermented brown algae extract (*Sargassum* sp.) on *Zea mays* L. to test the effect on corn growth. Parameters such as the height, stem circumference, cob length, and diameter improved after spraying with algal extracts compared with the control and urea-phosphate-potassium fertilizer application. Baroud et al. [147] tested the effects of brown algal extracts (*C. gibraltarica*, *F. spiralis*, and *Bifurcaria bifurcate*) on the germination, growth, and biochemical profile of tomatoes. Improvements in the germination rate and seedling biomass were observed. Compared with the control, the biochemical composition was higher in terms of protein content (21.59 mg/g to 54.64 mg/g), pigments (0.38 mg/g to 0.61 mg/g), and polysaccharide content (21.04 mg/g to 56.38 mg/g). Brown algae were also used to improve the quality of postharvest products. Extracts of brown algae *Sargassum crassifolium*, *S. cristaefolium*, *S. aquifolium*, and *Turbinaria murayana* were used as sprays for tomatoes. More fruits (15 fruits/plant) were reported when algae were added to the urea than in the control (9 fruits/plant). In addition to harvest and storage, tomatoes sprayed with brown algae had better texture after 7 days of storage at room temperature [148].

## 5. Future Directions

The bioactivity of algal carotenoids and the increasing awareness of their potential health-promoting properties make them attractive for application in various industries and fields, including nutraceuticals, food, feed, pharmaceuticals, and cosmetics [53]. The global market for carotenoids is expected to grow from an estimated USD 1.5 billion in 2019 to USD 2.0 billion in 2026 [91]. Algae production is still concentrated in Asian countries, with China dominating with a total production of over 56% of global aquaculture [149]. Microalgae are already widely used for the commercial production of carotenoids, and their use is rapidly increasing in various sectors due to their fast growth rate, resource sustainability, significantly higher production of carotenoids compared with macroalgae or terrestrial plants, and ability to quickly adapt to new or changing growth conditions [20]. Macroalgae also produce more biomass than higher plants (they grow more than ten times faster) and can be grown in both fresh and marine water, and their cultivation is carried out without the use of pesticides and/or antibiotics, etc. [149], which in turn highlights their industrial potential for isolating valuable constituents. Therefore, future research in this field, especially to develop new technologies to improve the efficiency of algae extraction, is needed.

Accordingly, the production of carotenoids from algae has a bright future, but there are still some major challenges that need to be overcome, mainly related to the cost of algae production, optimization of harvesting and extraction of key compounds, and stability and storage of the isolated products. Also, the current issues of global warming and sea level rise are negatively impacting algal biomass production and quality, leading to losses and degradation of beneficial target components. The numerous current investigations in this scientific field are an indication that this industry is growing exponentially and will certainly lead to more competitive processes and final products with a wide range of applications.

## Data Availability

No new data were created or analyzed in this study. Data sharing is not applicable to this article.
